# Investigating the impact of oligo-chitosan on the growth dynamics and yield traits of *Oryza sativa* L. ‘BRRI dhan29’ under subtropical conditions

**DOI:** 10.1016/j.heliyon.2024.e41552

**Published:** 2024-12-28

**Authors:** Afrina Rahman, Rayhan Ahammed, Jayanta Roy, Md Liton Mia, Mohammad Abdul Kader, Mubarak A. Khan, Md Harun Rashid, Uttam Kumer Sarker, Md Romij Uddin, Md Shafiqul Islam

**Affiliations:** a Department of Agronomy, Bangladesh Agricultural University, Mymensingh, 2202, Bangladesh; b Department of Biotechnology, Bangladesh Agricultural University, Mymensingh, 2202, Bangladesh; c Department of Arts & Sciences, Ahsanullah University of Science and Technology, Dhaka, 1208, Bangladesh; d Former Director General, Bangladesh Atomic Energy Commission and Scientific Advisor, BJMC, Ministry of Jute and Textile, Dhaka, 1000, Bangladesh

**Keywords:** Polysaccharide, Chitosan, Bio-stimulant, Rice, Yield

## Abstract

Reducing the harmful chemical use along with obtaining potential yield in field is a worth exploring practice in rice cultivation. To mitigate the prevailing yield gap, the current study was designed to evaluate the effect of chitosan in improving growth, yield contributing characters and yield of rice. The experiment comprised eight different treatments *viz.* control (no fertilizer and Chitosan) (T_0_), conventional method (with fertilizers) (T_1_), conventional method with foliar spray of 100 ppm chitosan solution (T_2_), conventional method with foliar spray of 300 ppm chitosan solution (T_3_), conventional method with foliar spray of 500 ppm chitosan solution (T_4_), only foliar spray of 100 ppm chitosan solution (T_5_), only foliar spray of 300 ppm chitosan solution (T_6_), and only foliar spray of 500 ppm chitosan solution (T_7_). The experiment was laid out in a completely randomized block design containing three replications. Data on different vegetative and yield contributing characters were recorded to evaluate the treatments effectiveness in improving rice yield. Different growth and yield contributing characters showed significant improvement after applying chitosan in addition to the conventional production system. The conventional method with foliar spray of 500 ppm chitosan solution had a greater positive effect on yield contributing characters and yield. In vegetative characters, the highest plant height became (87.3 cm), number of tiller hill^−1^ (13.7), Total dry matter (12.9), leaf area index (1.35), and chlorophyll content (57.73). On the basis of assessed treatments in yield contributing characters and yield, the highest plant height was (91.8 cm), no. of grains panicle^−1^ (145.29), grain yield (6.37 t ha^−1^), straw yield (6.47 t ha^−1^). Results showed that the conventional method with foliar spray of different concentration of chitosan solution was able to increase yield up to 26 % in comparison to the conventional method. Overall, our findings suggest that additional foliar spray of chitosan with previously recommended cultivation practice can increase the yield per unit area and offers promising technology to achieve potential yield in farmer's field.

## Highlights

• Chitosan raised LAI, TDM, and chlorophyll in BRRI dhan29.
• Traditional 500 ppm chitosan foliar application increased BRRI dhan29 production.
• Rice yield rose 26 % with conventional foliar spray of varied chitosan concentrations.

## Graphical abstract

**Figure:**
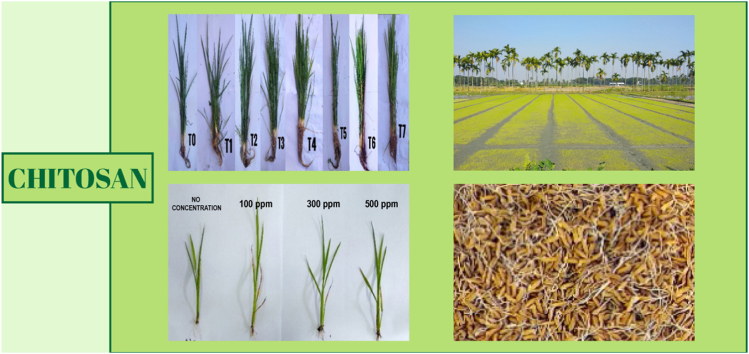

## 1 Introduction

Rice (*Oryza sativa*) is considered as one of the nutritious cereal grains of today's world which assists as the primary source of carbohydrate for more than half of the world's population [1]. Among cereals, rice protein ranked high in nutritional quality and provides 15 % of global human per capita protein [2]. Additionally, it also provides minerals, vitamins, and fiber to human diet. In Bangladesh, rice is the foremost cultivated crop that contributes 95 % of the annual food grain production and supplies 75 % of the carb and 50 % of the total protein intake of an average person in the country [3]. Bangladesh's agronomic and geographic characteristics are ideal for rice farming. There are three different types of rice in Bangladesh namely *aus*, *aman*, and *boro*, depending on the time of year they are grown. The genus Oryza has twenty-one wild species and two cultivated species [4]. In 2022, Bangladesh ranked 3rd among the 118 rice producing countries of the world by producing around 38 million tons of rice in 11 million hectares of cultivated area [5,6]. Bangladesh has been facing ongoing challenges in achieving food security. In June 2020, official estimates showed that 30 % of Bangladesh's population was impoverished, up from 21 % in June 2019. The pandemic caused income loss and a decline in remittances, which contributed to an increase in poverty. In addition, the country experienced frequent floods in 2020, which damaged households and the agricultural sector and exacerbated food insecurity [7]. The government was able to export only 11297 tons of rice in 2021 after meeting the domestic requirement. Bangladesh has an excellent sub-tropical climate conducive for rice production, although the average yield (4.8 t ha^−1^) is very poor and the country ranked 37 among the rice producing countries [8]. Moreover, continuing decrease of rice cultivation land and increasing population is creating further pressure to increase rice yield to meet the growing food demand of the country.

Currently, rice growers are employing different conventional approaches along with a large quantity of synthetic fertilizer in the rice field to increase yield. The aim of producing more by applying excessive fertilizer caused deterioration of soil chemical, physical, and biological properties, thus contributing to the increase of soil and water pollution. Recent report stated that rice yield has stagnated and even declined in some cases despite of using high levels of fertilizer and high yielding varieties [[9], [10], [11]]. The results encourage the researchers to utilize unconventional methods like genetic engineering, biotechnology, or the application of plant hormones to reduce the gap between rice production potential and actual yield [12]. Application of biostimulants plays an important role in shifting towards a more sustainable production system. Plant biostimulants are considered as any substances or microorganisms that are applied to plants with the aim to enhance nutrition and water uptake efficiency, activation of natural metabolic mechanisms against stress and/or improve crop quality, regardless of its nutrients content [13]. In plants, biostimulants can be used alone or in combination with other substances or microbes. In rice, a small concentration of biostimulants application has the capacity to positively alter the major physiological processes including photosynthesis, plant nutrition uptake, and respiration which influences the quality and quantity of the desired products [14]. Recently, it has been used in agriculturally developed counties on a wide range of crops like soybean, spinach, tomato to cope up plants to survive under stress conditions and improve yield. The current market for plant biostimulants is increasing and it is expected to grow over 4 billion by 2025 [15]. Humic and falvic acids, protein hydrolysates, seaweed extracts and botanicals, chitosan and other biopolymers, inorganic compounds, beneficial fungi and bacteria are the major categories of biostimulants. Among these different groups of product technologies, it has been shown that additional supply of chitosan has the potential to improve growth and yield of crops [16].

Oligochitosan is one of the most effective oligosaccharins which is obtained by hydrolysis or degradation of chitosan. It consists of β-1, 4-linked 2-amino-D-glucose units and contains only small amounts of 2-acetamido-D-glucose units. Many researchers have identified that oligochitosan is water-soluble, biocompatible, and possesses versatile functional properties [17]. Oligochitosan has the ability to inhibit pathogen invasion, induce phytoalexins production, and excite defense-related genes expression [18]. It can activate plant innate immunity on rice [19], grapevine [20], tobacco [9], oilseed rape [21]. Chitosan is a well-studied polysaccharide derived from chitin, which occurs in the exoskeletons of shellfish like shrimp, lobster, and crabs, as well as in the cell walls of fungi [22]. Chitosan is well accepted in different countries for its non-toxic, cheap, and biodegradable nature. In Bangladesh, chitosan can be easily produced from shrimp waste. Every year Bangladesh produces around 23190.24 tons of industrial shrimp waste which can be a great source of producing cheap chitosan inside the country and use it for the welfare of agricultural production [23] by reducing harmful chemical use in agricultural production systems. This polysaccharide is widely used as a plant growth and development enhancer. Nowadays, there is a rise in the usage of chitosan and humic acid as natural stimulants. They have the unique qualities of being readily biodegradable and environmentally benign. As a liquid plant growth enhancer, humic acid is a suspension based on potassium humates that can be sprayed straight into plant foliage [24]. By influencing processes related to water and food intake, protein synthesis, photosynthesis, cell respiration, and enzyme activity, it promotes plant growth [25]. Plant immune systems are regulated by chitosan, which also causes resistant enzymes to be excreted. It has significant benefits on agriculture, including helping plants' roots absorb more nutrients from the soil, increasing the conversion of organic matter into inorganic nutrients, and providing bacteria in the soil with a source of carbon [26]. Chitosan is broken down by soil microorganisms and then taken up by the root. In agriculture, chitosan application may increase microbial population even in the absence of chemical fertilizer [27]. There are several uses for chitosan. It is safe for both humans and animals to use due to its strong affinity and lack of toxicity [28].

Application of chitosan also increases the yield by protecting against microorganisms through the upregulation of multiple defense-enzymes [29,30]. Compared to the untreated control, the chitosan-treated plant had a noticeably higher number of branches [31]. Chitosan is mostly utilized in agriculture as an environmentally friendly bio-pesticide that increases plants' natural defenses against fungal infections, as well as a natural seed treatment and plant growth promoter [32]. Higher chitin content plants are more resistant to disease [33]. Additionally, beneficial association among chitosan and microbes can improve the nutrient uptake efficiency [34]. In plants, chitosan elicits numerous defense responses related to biotic and abiotic stresses [35]. It has been utilized effectively in many plants related applications to increase plant productivity as well as protect plants against the attack of pathogens [36]. Previous studies revealed that chitosan has a potential to enhance plant growth as well as increase yield in many crops including apple, wheat, maize and rice [[37], [38], [39], [40]]. Despite of having so many beneficial effects of chitosan on crops, limited research work has been done on the effect of chitosan on rice.

In Bangladesh, the rice sector not only stands out for economic importance but also for its relevance as 48 % rural employment generator [3]. Therefore, any action intended for the betterment of the sector would have a direct impact at all levels of the country. Keeping this in mind, the present research work was designed to study the effect of chitosan on growth, yield attributes, and yield of rice.

## 2 Materials and methods

### 2.1 Experimental site and plant material

The experiment was conducted at the Agronomy Field Laboratory, Bangladesh Agricultural University (BAU), Mymensingh from November 2019 to April 2020 to identify the effect of oligo-chitosan on the growth and yield of rice in bioclimatic condition of Bangladesh. In this study, we selected a single *boro* rice cultivar i.e., BRRI dhan29, a high yielding and widely cultivated cultivar in Bangladesh [41]. The research farm was sited at 24°75 N latitude and 90°50 E longitude with an elevation of 18 m which belongs to the Old Brahmaputra Floodplain (AEZ-9) [42]. The soil of the research field belongs to the Sonatola series of non-calcareous dark grey floodplain soil types under AEZ-9. The land had a silty loam texture and was classified as medium-high. Soil's physical and chemical properties of the experimental site is displayed in Table 1 [43]. Table 2 shows the research period's agro-climatic conditions.

**Table 1:** The physical, and chemical characteristics of the experimental field.

Physical properties of the initial soil (0–15 cm depth)	
Constitution	Results

Sand (%) (0.0–0.02 mm)	20
Silt (%) (0.02–0.002 mm)	67
Clay (%) (<0.002 mm)	13
Soil textural class	Silt Loam
Particle density (g/cc)	2.60
Bulk density (g/cc)	1.35
Porosity (%)	46.67

**Chemical characteristics of soil**	

**Constitutions**	**Results**

pH	6.80
Organic matter (%)	1.29
Nitrogen (%)	0.101
Phosphorous (ppm)	26.00
Potassium (me %)	0.14

**Table 2:** Monthly record of temperature, relative humidity, rainfall and sunshine during the period from November 2019 to April 2020.

Month and year	Air temperature (^0^C)			Rainfall (mm)	Relative humidity (%)	Sunshine (hrs.)
	Maximum	Minimum	Average			
November 2019	30.2	19.50	24.85	2.00	83.4	214.9
December 2019	24.5	13.4	19.2	1.6	82.7	160.8
January 2020	23.46	12.61	18.05	2.1	84.12	120.4
February 2020	26.17	14.23	20.20	0.00	75.03	164.4
March 2020	30.31	18.85	24.58	26.9	73.61	224.9
April 2020	31.45	21.49	26.47	106.7	78.26	185.7

### 2.2 Description of the variety

BRRI dhan29 was used as the test crop in this experiment which is one of the most cultivated rice varieties in Bangladesh. This variety was developed at the Bangladesh Rice Research Institute from the cross between BG 90-2 and BR 51-46-5 in 1994. It is recommended for *boro* season. The average plant height of the variety is 90–95 cm at the ripening stage. The grains are medium-fine and white. It is highly resistant to rice tungro virus (RTV), bacterial leaf blight (BLB), stem rot, and blast. It requires about 155–160 days to complete its life cycle with an average grain yield of 5.0–5.5 t ha^−1^.

### 2.3 Preparation of chitosan spray solution for foliar spray

Prawn shell waste was used to extract chitosan in the laboratory by deacetylation process described by Ref. [30]. A dose of 3.2 kGy per hour generated from a 120 k Curie radiation source was applied to obtain a high molecular weight chitosan solution (molecular weight = 83 KD, deacetylation degree 82.7 %, viscosity less than 200 mPa-s, 2 % acetic acid in 55 °C). Laboratory grade chemicals were used for the preparation of chitosan solution. Finally, 40 kGy gamma irradiated chitosan solution was selected based on initial observatory test on the rice seedlings in laboratory.

### 2.4 Experimental treatments and design

Experiments were designed using a total of eight treatments. The treatments consists of a combination of different concentrations of chitosan solutions and management practices such as control (no fertilizer and Chitosan) (T_0_), conventional method (with fertilizers) (T_1_), conventional method with foliar spray of 100 ppm chitosan solution (T_2_), conventional method with foliar spray of 300 ppm chitosan solution (T_3_), conventional method with foliar spray of 500 ppm chitosan solution (T_4_), only foliar spray of 100 ppm chitosan solution (T_5_), only foliar spray of 300 ppm chitosan solution (T_6_), and only foliar spray of 500 ppm chitosan solution (T_7_).

The field research was laid out in a randomized complete block design (RCBD) with three replications. The total number of plots was 24 and the size of each plot was 5 m^2^ (2.5 m in length × 2.0 m in width). The distance between block to block and plot to plot was maintained at 1.0 m and 0.5 m, respectively.

### 2.5 Cultivation practices

Rice seedlings were raised by dividing the nursery bed into four parts to maintain the different chitosan concentration (100 ppm, 300 ppm, 500 ppm) treatment seedling and without chitosan (control) treatment seedling. The sprouted seeds were sown in the nursery bed and 55 days old seedlings were uprooted to transplant in the main experimental field. The main field was tilled using a power tiller with four times ploughing and cross ploughing followed by laddering. Following the treatment specification, required plots were fertilized with recommended dose of urea, muriate of potash (MoP), triple super phosphate, gypsum, and zinc sulphate at 300, 100, 120, 60, and 10 kg ha^−1^, respectively [44]. Entire amounts of TSP, MoP, gypsum, and zinc sulphate were applied to the plot during final land preparation whereas urea was applied in three equal installments at 15, 30 and 45 days after transplanting (DAT). Seedlings were transplanted maintaining a spacing of 25 cm × 15 cm at a rate of two seedlings per hill. Different concentration of chitosan solution was prepared at the day of spraying and 200 ml of chitosan was sprayed to each plot according to the treatment specification. Chitosan was sprayed during the early morning at 15 days interval starting from 15 days after germination to end of seed set. Intercultural operations were performed as and when necessary. No chemical was used for pest or weed control. The matured crops (80–90 % of the panicles turned into golden yellow color) were harvested and then the grains and straws were sun dried. After cleaning, the grain and straw yield plot^−1^ were recorded and converted to t ha^−1^.

### 2.6 Data collection

Five hills (excluding border hills) from each plot were randomly selected and marked with a sample card. During vegetative stage, different growth dynamics such as plant height (PH), total dry matter (TDM), chlorophyll content, number of tillers hill^−1^ (TT), and leaf area index (LAI) were recorded from selected plants at 30, 50, and 70 DAT, respectively. Chlorophyll content (SPAD) was measured from a fully expanded leaf using a Minolta 502+ SPAD meter (Konica Minolta, Gainesville, FL). To determine LAI, a hill was uprooted, and the leaf blades were alienated from the leaf sheath. Leaf area was measured by a leaf area meter (LI 3100, Licor, Inc., Lincoln NE, USA) and LAI was calculated using the standard formula described by Refs. [45,46]. After measuring the leaf area, the same plant samples were dried in an electric oven for 72 h to record the dry matter content.

At harvest, data were collected on plant height (cm), number of total tillers hill^−1^, number of effective tillers hill^−1^, non-effective tillers hill^−1^, panicle length (cm), number of grains panicle^−1^, number of sterile grains panicle^−1^, 1000-grain weight (g), grain yield (t ha^−1^), straw yield (t ha^−1^), and harvest index (%).

### 2.7 Data collection procedure

#### 2.7.1 Plant height (cm) at harvest

Measurement of panicle length was taken from the basal node of the rachis to the apex of each panicle. Each observation was an average of 5 panicles.

#### 2.7.2 Effective tillers hill^−1^ (no.)

The panicles which had at least one grain were considered effective tillers. The number of effective tillers hill^−1^ was recorded and finally averaged for counting effective tillers number hill^−1^.

#### 2.7.3 Non-effective tillers hill^−1^ (no.)

The tiller having no panicle was regarded as non-effective tillers. The number of non-effective tillers hill^−1^ was recorded and finally averaged for counting non-effective tillers number.

#### 2.7.4 Panicle length^−1^ (cm)

Measurement of panicle length was taken from the basal node of the rachis to the apex of each panicle. Each observation was an average of 5 panicles.

#### 2.7.5 Grains panicle^−1^ (no.)

The number of filled grains panicle^−1^ plus the number of unfilled grains panicles gave the total number of grains panicle^−1^.

#### 2.7.6 1000-Grain weight (g)

One thousand cleaned dried grains were counted randomly from each sample and weighed by using a digital electric balance at the stage the grain retained about 12 % Moisture and the mean weights were expressed in grams.

#### 2.7.7 Grain yield (t ha^−1^)

Grain yield was determined from the central 1 m^2^ areas of each plot and expressed as (t ha^−1^) on about a 12 % moisture basis. Grain moisture content was measured by using a digital moisture tester.

#### 2.7.8 Straw yield (t ha^−1^)

The straw yield was determined from the central 1 m^2^ areas of each plot. After separating of grains, the sub-samples were oven-dried to a constant weight and finally converted to (t ha^−1^).

#### 2.7.9 Biological yield (t ha^−1^)

Grain yield and straw yield were all together regarded as biological yield. The biological yield was calculated with the following formula:

Biological yield (t ha^−1^) = Grain yield (t ha^−1^) + Straw yield (t ha^−1^)

#### 2.7.10 Harvest index (%)

It denotes the ratio of grain yield to biological yield and was calculated with the following formula.

$$\text{Harvest}\;\text{index}\;\left(\text{HI}\right)=\frac{Grain\;yield}{Biological\;yield}x\;100$$

### 2.8 Statistical analysis

The recorded data were compiled and tabulated for statistical analysis. Analysis of variance (ANOVA), box plot, PCA, correlation, and heatmap analyses were performed using the statistical package ‘R [47]. All data were subjected to one-way ANOVA. The averages of treatment means were compared by one-way ANOVA followed by Tukey's post hoc test (*p* = 0.05).

## 3 Results

### 3.1 Effect of chitosan on growth parameters

#### 3.1.1 Plant height and total tiller

**Fig. 1:**
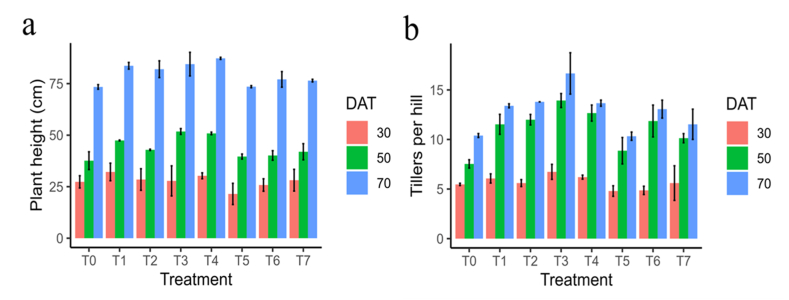
Effect of chitosan treatments on plant height (a), and total tiller hill^−1^ (b) of BRRI dhan29 at different DAT. Means with the same letters are not statistically different from each other (In plant height and total tiller, *P* < 0.0001 at 50 and 70 DAT, at 30 DAT *P* = 0.2764 and 0.0767 respectively). Control (no fertilizer and Chitosan) (T_0_), conventional method (with fertilizers) (T_1_), conventional method with foliar spray of 100 ppm chitosan solution (T_2_), conventional method with foliar spray of 300 ppm chitosan solution (T_3_), conventional method with foliar spray of 500 ppm chitosan solution (T_4_), only foliar spray of 100 ppm chitosan solution (T_5_), only foliar spray of 300 ppm chitosan solution (T_6_), and only foliar spray of 500 ppm chitosan solution (T_7_).

The analysis of variance (ANOVA) indicated that all vegetative growth parameters i.e., PH, and TT had a significant effect on rice plant vegetative growth establishment at 50, and 70 DAT, while non-significant on 30 DAT. The PH and TT hill^−1^ at 50 and 70 DAT was significantly influenced by chitosan application. The conventional method with foliar spray of 300 ppm chitosan solution treatment showed highest PH (51.8 cm) at 50 DAT, which is statistically different from the control. At 70 DAT, PH was the highest (87.3 cm) for conventional method with foliar spray of 500 ppm chitosan solution treatment which is statistically similar to T_2_, T_3_, and T_4_ but different to control plants. The lowest PH (37.6 cm and 73.4 cm) was recorded in control treatment for both data collection dates (Fig. 1a). Likewise, in the conventional method with foliar spray of 500 ppm chitosan solution, the highest tillers hill^−1^ at 50 and 70 DAT were 12.7 and 13.7, respectively. The lowest tiller producers for 50 DAT and 70 DAT were 7.5 and 10.3 for the rice plants grown under control and only 100 ppm chitosan solution treatment conditions, respectively (Fig. 1b).

#### 3.1.2 Chlorophyll content, leaf area index, and total dry matter

**Fig. 2:**
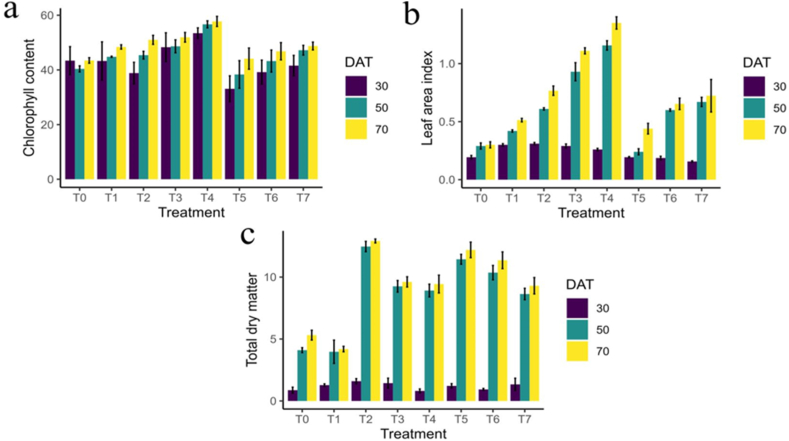
Effect of chitosan treatments on chlorophyll content (a), leaf area index (b), and total dry matter (c) of BRRI dhan29 at different DAT. Means with the same letters are not statistically different from each other (In leaf area index, *P* < 0.0001. In chlorophyll content and total dry matter, *P* < 0.0001 at 50 and 70 DAT, at 30 DAT *P* = 0.0032 and 0.0218 respectively).Control (no fertilizer and Chitosan) (T_0_), conventional method (with fertilizers) (T_1_), conventional method with foliar spray of 100 ppm chitosan solution (T_2_), conventional method with foliar spray of 300 ppm chitosan solution (T_3_), conventional method with foliar spray of 500 ppm chitosan solution (T_4_), only foliar spray of 100 ppm chitosan solution (T_5_), only foliar spray of 300 ppm chitosan solution (T_6_), and only foliar spray of 500 ppm chitosan solution (T_7_).

The influence of chitosan on chlorophyll content, LAI and TDM can be showed in Fig. 2. All three of them showed significant effects (*P* ≤ 0.05). Rice plants treated with the combining conventional method with 500 ppm foliar spray of chitosan solution showed an increase of about 29 % and 21 % chlorophyll content than control and conventional method, respectively at 50 DAT, which was 25 % and 16 % greater at 70 DAT (Fig. 2a). This finding indicates that chitosan can significantly increase rice plant capacity to photosynthesize and accumulate organic matter, both of which are beneficial for the growth of young plants. At 50 and 70 DAT, plants sprayed with chitosan solution showed increased LAI compared to conventional method and control. In both cases, T_4_ treatment had the highest LAI (Fig. 2b). In the plants that were sprayed with chitosan, similar trends were also observed. The TDM content was higher for the combined application of fertilizer and chitosan solution (T_2_-T_4_) than the control and sole application of fertilizer (T_1_) and chitosan solution (T_5_-T_7_). At 50 and 70 DAT, the highest TDM at 50 DAT and 70 DAT were 12.5 and 12.9, respectively, recorded in conventional method with foliar spray of 500 ppm chitosan solution and the lowest 4.0 and 4.2 TDM for 50 DAT and 70 DAT, respectively, were measured in control treatment (Fig. 2c).

**Fig. 3:**
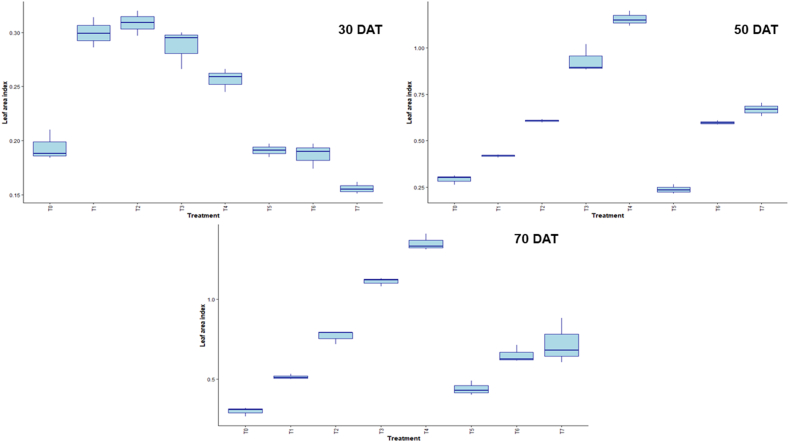
Effect of chitosan treatments on leaf area index of BRRI dhan29 at different DAT. Means with the same letters are not statistically different from each other (In leaf area index, *P* < 0.0001).Control (no fertilizer and Chitosan) (T_0_), conventional method (with fertilizers) (T_1_), conventional method with foliar spray of 100 ppm chitosan solution (T_2_), conventional method with foliar spray of 300 ppm chitosan solution (T_3_), conventional method with foliar spray of 500 ppm chitosan solution (T_4_), only foliar spray of 100 ppm chitosan solution (T_5_), only foliar spray of 300 ppm chitosan solution (T_6_), and only foliar spray of 500 ppm chitosan solution (T_7_).

**Fig. 4:**
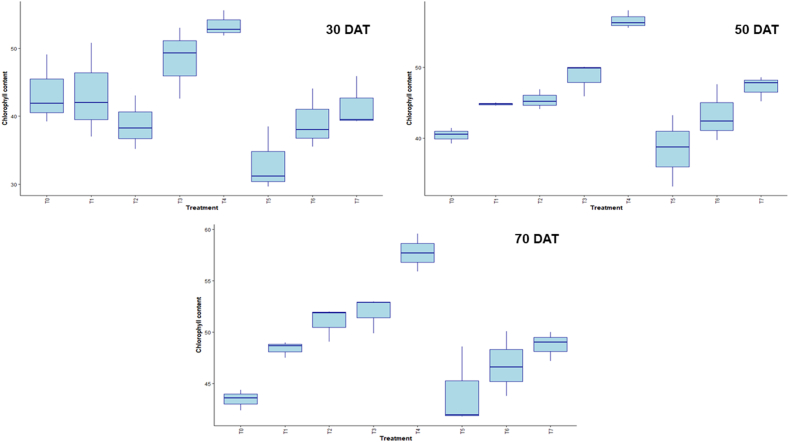
Effect of chitosan treatments on chlorophyll content of BRRI dhan29 at different DAT. Means with the same letters are not statistically different from each other (In chlorophyll content, *P* < 0.0001 at 50 and 70 DAT, and at 30 DAT *P* = 0.0032).Control (no fertilizer and Chitosan) (T_0_), conventional method (with fertilizers) (T_1_), conventional method with foliar spray of 100 ppm chitosan solution (T_2_), conventional method with foliar spray of 300 ppm chitosan solution (T_3_), conventional method with foliar spray of 500 ppm chitosan solution (T_4_), only foliar spray of 100 ppm chitosan solution (T_5_), only foliar spray of 300 ppm chitosan solution (T_6_), and only foliar spray of 500 ppm chitosan solution (T_7_).

**Fig. 5:**
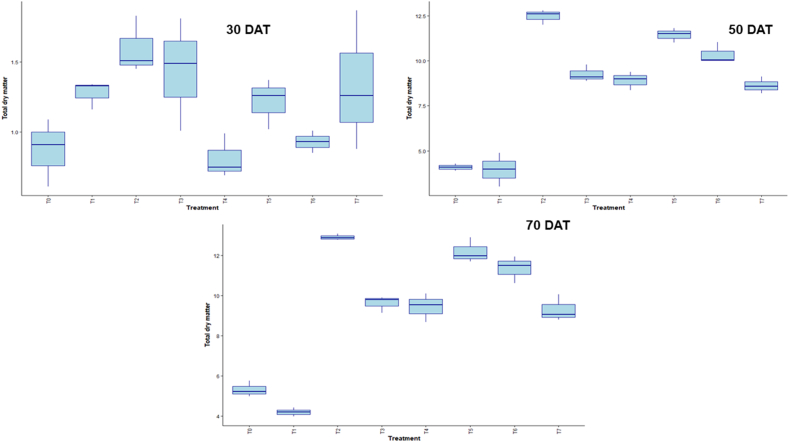
Effect of chitosan treatments on total dry matter of BRRI dhan29 at different DAT. Means with the same letters are not statistically different from each other (In total dry matter, *P* < 0.0001 at 50 and 70 DAT, and at 30 DAT *P* = 0.0218).Control (no fertilizer and Chitosan) (T_0_), conventional method (with fertilizers) (T_1_), conventional method with foliar spray of 100 ppm chitosan solution (T_2_), conventional method with foliar spray of 300 ppm chitosan solution (T_3_), conventional method with foliar spray of 500 ppm chitosan solution (T_4_), only foliar spray of 100 ppm chitosan solution (T_5_), only foliar spray of 300 ppm chitosan solution (T_6_), and only foliar spray of 500 ppm chitosan solution (T_7_).

Box plots of the LAI at 30, 50, and 70 days after treatment (DAT) for various treatments (T_0_-T_7_) are displayed in the figure. Over time, LAI rises, with T_3_ typically showing greater values at all time intervals, particularly at 50 DAT (Fig. 3). Fig. 4 displays boxplots of chlorophyll content at three time points: 30, 50, and 70 days after treatment (DAT) for each of the treatments (T_0_–T_7_). Chlorophyll content varies more from treatment to treatment, with some showing significant changes over time. The figure shows boxplots of TDM for treatments (T_0_–T_7_) at 30, 50, and 70 days after treatment (DAT). The TDM increases with time, and at later stages, some treatments show higher variations and accumulation (Fig. 5).

### 3.2 Comparative results on rice yield contributing characters and yield influenced by chitosan application

Different yield contributing characters of rice differed significantly (*p* ≤ 0.05) due to treatment differences except TT, NET, PL, SGP, HI (Table 3).

**Table 3:** Effect of chitosan on yield contributing characters of BRRI dhan29.

Treatments	Total tiller hill^−1^ (no.)	Non-effective tiller hill^−1^ (no.)	Panicle length (cm)	Sterile grains panicle^−1^ (no.)	Harvest index (%)
T_0_	10.8	1.27	23.19	11.57	51.69
T_1_	13.26667	1.53	23.58	11.26	48.48
T_2_	13.93333	1.8	23.87	10.97	48.35
T_3_	13.33333	1.07	23.83	10.63	48.9
T_4_	12.46667	0.93	23.36	10.44	49.61
T_5_	10.8	1.47	22.89	10.88	48.24
T_6_	11.43333	1	22.95	11.01	48.32
T_7_	12.43333	1.1	22.99	10.77	49.49
CV	15.2728	33.35	2.32	4.74	3.69
P value	0.3531	0.22	0.21	0.2692	0.3442

Control (no fertilizer and Chitosan) (T_0_), conventional method (with fertilizers) (T_1_), conventional method with foliar spray of 100 ppm chitosan solution (T_2_), conventional method with foliar spray of 300 ppm chitosan solution (T_3_), conventional method with foliar spray of 500 ppm chitosan solution (T_4_), only foliar spray of 100 ppm chitosan solution (T_5_), only foliar spray of 300 ppm chitosan solution (T_6_), and only foliar spray of 500 ppm chitosan solution (T_7_).

#### 3.2.1 Plant height, effective tiller, grain per panicle, and 1000-grain weight

**Fig. 6:**
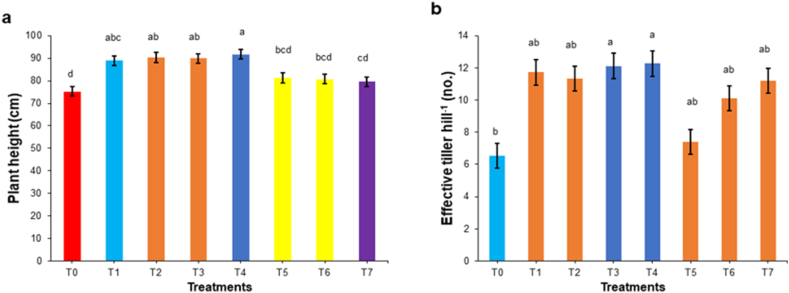
Effect of chitosan treatments on plant height (a) and effective tiller per hill (b) of BRRI dhan29. Means with the same letters are not statistically different from each other (In plant height, *P* = 0.0002 and effective tiller, *P* = 0.0104).Control (no fertilizer and Chitosan) (T_0_), conventional method (with fertilizers) (T_1_), conventional method with foliar spray of 100 ppm chitosan solution (T_2_), conventional method with foliar spray of 300 ppm chitosan solution (T_3_), conventional method with foliar spray of 500 ppm chitosan solution (T_4_), only foliar spray of 100 ppm chitosan solution (T_5_), only foliar spray of 300 ppm chitosan solution (T_6_), and only foliar spray of 500 ppm chitosan solution (T_7_).

**Fig. 7:**
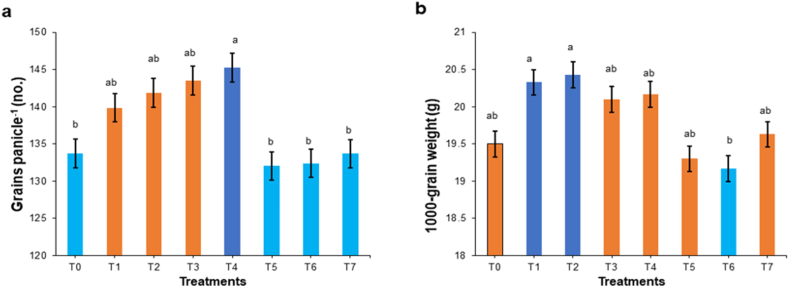
Effect of chitosan treatments on grains per panicle (a) and 1000-grain weight (b) of BRRI dhan29. Means with the same letters are not statistically different from each other (In grains per panicle, *P* = 0.0027 and 1000-grain weight, *P* = 0.0078).Control (no fertilizer and Chitosan) (T_0_), conventional method (with fertilizers) (T_1_), conventional method with foliar spray of 100 ppm chitosan solution (T_2_), conventional method with foliar spray of 300 ppm chitosan solution (T_3_), conventional method with foliar spray of 500 ppm chitosan solution (T_4_), only foliar spray of 100 ppm chitosan solution (T_5_), only foliar spray of 300 ppm chitosan solution (T_6_), and only foliar spray of 500 ppm chitosan solution (T_7_).

Results of yield trial showed that application of chitosan noticeably improved PH, ET, GPP, 1000-grains weight (TGW). Rice plants treated with a combination of 500 ppm chitosan and conventional method resulted the highest PH (91.8 cm) (Fig. 6a), ET (12.3) (Fig. 6b), GPP (145.3) (Fig. 7a), while TGW was the highest (20.43g) at T_2_ (conventional method with foliar spray of 100 ppm chitosan) (Fig. 7b). These values are higher than the single use of conventional method and control treatment. Statistically similar results were also noted for treatments using 100 and 300 ppm chitosan solution when combined with the conventional method. The conventional method with 500 ppm chitosan spray yielded the lowest NET (0.93) and SGP (10.4) numerically, however the fact that no statistically significant difference was recorded.

#### 3.2.2 Grain yield and straw yield

**Fig. 8:**
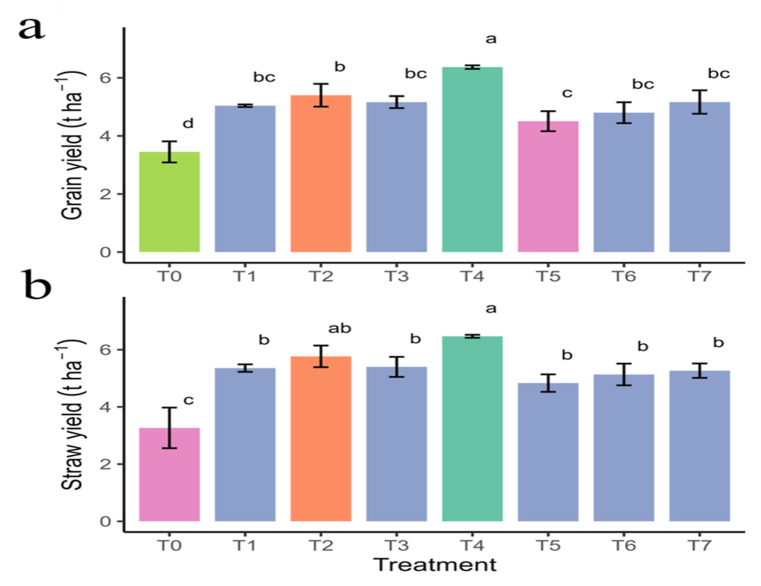
Effect of chitosan treatments on grain yield (a), and straw yield (b) of BRRI dhan29. Means with the same letters are not statistically different from each other (*P* < 0.0001).Control (no fertilizer and Chitosan) (T_0_), conventional method (with fertilizers) (T_1_), conventional method with foliar spray of 100 ppm chitosan solution (T_2_), conventional method with foliar spray of 300 ppm chitosan solution (T_3_), conventional method with foliar spray of 500 ppm chitosan solution (T_4_), only foliar spray of 100 ppm chitosan solution (T_5_), only foliar spray of 300 ppm chitosan solution (T_6_), and only foliar spray of 500 ppm chitosan solution (T_7_).

Moreover, the findings show that chitosan can effectively increase rice yield. The rice plants grown using a combination of the conventional method and a 500-ppm chitosan spray produced the maximum yield (6.4 t ha^−1^), which was almost 25 % higher than the conventional method and 45 % higher than the control treatment (Fig. 8a). More interestingly, rice plants sprayed with only 500 ppm chitosan solution yielded ∼2 % higher than the conventional method which clearly indicated the effectiveness of chitosan in improving rice yield. Similar trends were also observed for rice SY trait as well (Fig. 8b). The results revealed that foliar spray of chitosan solution has a positive effect on different yield contributing traits and yield of rice. Moreover, the effects were profound when conventional method was combined with 300 ppm and 500 ppm foliar spray of chitosan solutions treatments than the single use of conventional and control treatments.

### 3.3 PCA, correlation, and heat map analysis of growth, yield and yield contributing traits in rice under different treatment

#### 3.3.1 Principal component analysis

**Fig. 9:**
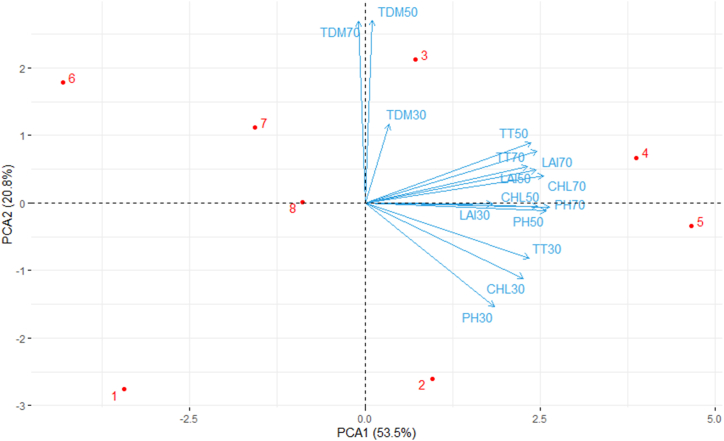
Bi-plot of principal component analysis (PCA) showing the first two principal components (PC 1 and PC 2) on growth traits For PCA, data on weed growth, yield and yield contributing attributes under residues. The red dots are indicating five residue treatments, like 1 = Control (no fertilizer and Chitosan), 2 = conventional method (with fertilizers), 3 = conventional method with foliar spray of 100 ppm chitosan solution, 4 = conventional method with foliar spray of 300 ppm chitosan solution, 5 = conventional method with foliar spray of 500 ppm chitosan solution, 6 = only foliar spray of 100 ppm chitosan solution, and 7 = only foliar spray of 300 ppm chitosan solution, and 8 = only foliar spray of 500 ppm chitosan solution. Where PH30= Plant height at 30 DAT, PH50= Plant height at 50 DAT, PH70= Plant height at 70 DAT, TT30 = No. of total tiller hill^−1^ at 30 DAT, TT50 = No. of total tiller hill^−1^ at 50 DAT, TT70 = No. of total tiller hill^−1^ at 70 DAT, LAI30 = Leaf area index at 30 DAT, LAI50 = Leaf area index at 50 DAT, LAI70 = Leaf area index at 70 DAT, CHL30 = chlorophyll content at 30 DAT, CHL50 = chlorophyll content at 50 DAT, CHL70 = chlorophyll content at 70 DAT, TDM30 = Total dry matter at 30 DAT, TDM50 = Total dry matter at 50 DAT, TDM70 = Total dry matter at 70 DAT.

Principal Component Analysis was used to determine the traits that best describe the impact of oligo-chitosan on the growth of the widely cultivated variety, BRRI dhan29, in Bangladesh. The bi-plot of the first two principal components and the loadings of variables are presented in Fig. 9. The x-axis represents the first principal component (PC1), which accounts for 53.5 % of the total variance and the y-axis represents the second principal component (PC2), which accounts for 20.8 % of the total variance. The TT at 50 DAT, TT at 70 DAT, LAI at 70 DAT, LAI at 50 DAT, chlorophyll content at 70 DAT, chlorophyll content at 50 DAT, PH at 70 DAT, LAI at 30 DAT, PH at 50 DAT, TT at 30 DAT, chlorophyll content at 30 DAT, PH at 30 DAT variables had a strong positive correlation with PC1. TDM at 30, 50, 70 DAT are more aligned with PCA2. The TT at 50 DAT, TT at 70 DAT, LAI at 70 DAT, LAI at 50 DAT, chlorophyll content at 70 DAT, chlorophyll content at 50 DAT, PH at 70 DAT, LAI at 30 DAT, PH at 50 DAT, TT at 30 DAT, chlorophyll content at 30 DAT and PH at 30 DAT variables are closely aligned, indicating a strong positive correlation with each other. TDM at 30, 50, 70 DAT are cluster with each other that means they strong positive correlation with each other. Chlorophyll content at 50 DAT, PH at 70 DAT and LAI at 30DAT have no correlation with the TDM at 50 and 70 DAT. Chlorophyll content at 30 DAT and PH at 70 DAT have negative correlation with the TDM at 50 and 70 DAT. Treatment 3 is strongly influenced by PC2 and is distinct from other observations and treatments 4 and 5 are strongly influenced by PC1 and is distinct from other observations while treatment 7 is closer to the origin, indicating it has more average values for the principal components.

**Fig. 10:**
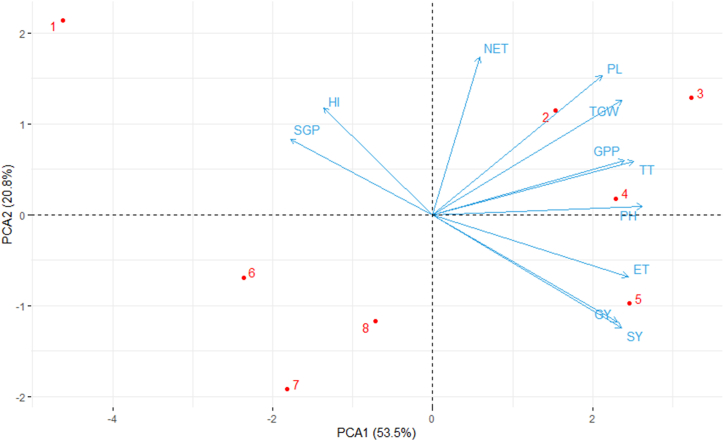
Bi-plot of principal component analysis (PCA) showing the first two principal components (PC 1 and PC 2) on yield traits For PCA, data on weed growth, yield and yield contributing attributes under residues. The red dots are indicating five residue treatments, like 1 = Control (no fertilizer and Chitosan), 2 = conventional method (with fertilizers), 3 = conventional method with foliar spray of 100 ppm chitosan solution, 4 = conventional method with foliar spray of 300 ppm chitosan solution, 5 = conventional method with foliar spray of 500 ppm chitosan solution, 6 = only foliar spray of 100 ppm chitosan solution, and 7 = only foliar spray of 300 ppm chitosan solution, 8 = and only foliar spray of 500 ppm chitosan solution. Where PH= Plant height (cm), TT = Total tiller hill^−1^, ET = Number of effective tillers hill^−1^, NET= Number of non-effective tillers hill^−1^, PL= Panicle length (cm), GPP= Number of grain panicle^−1^, SGP= Sterile grains panicle^−1^, TGW = 1000-grain weight (g), GY = Grain yield (t ha^−1^), SY= Straw yield (t ha^−1^), and HI= Harvest index (%).

Principal Component Analysis was used to determine the traits that best describe the impact of oligo-chitosan on the yield of the widely cultivated variety, BRRI dhan29, in Bangladesh. The bi-plot of the first two principal components and the loadings of variables are presented in Fig. 10. The x-axis represents the first principal component (PC1), which accounts for 53.5 % of the total variance and the y-axis represents the second principal component (PC2), which accounts for 20.8 % of the total variance. SY, BY, GY, TGW, GPP, PH, ET, and PL variables have a strong positive correlation with PC1. HI and SGP variables are closely aligned, indicating a strong positive correlation with each other. HI and SGP variables are clustered together and are more negatively correlated with the other group of variables such as SY, GY, and ET. SY, BY, GY, TGW, number of GPP, PH, ET, and PL are cluster that's mean they have strong positive correlation with each other. Treatment 8 is strongly influenced by PC2 and is distinct from other observations and treatment 4 is strongly influenced by PC1 and is distinct from other observations while treatment 1 and 2 are closer to the origin, indicating they have more average values for the principal components.

#### 3.3.2 Correlation analysis

**Fig. 11:**
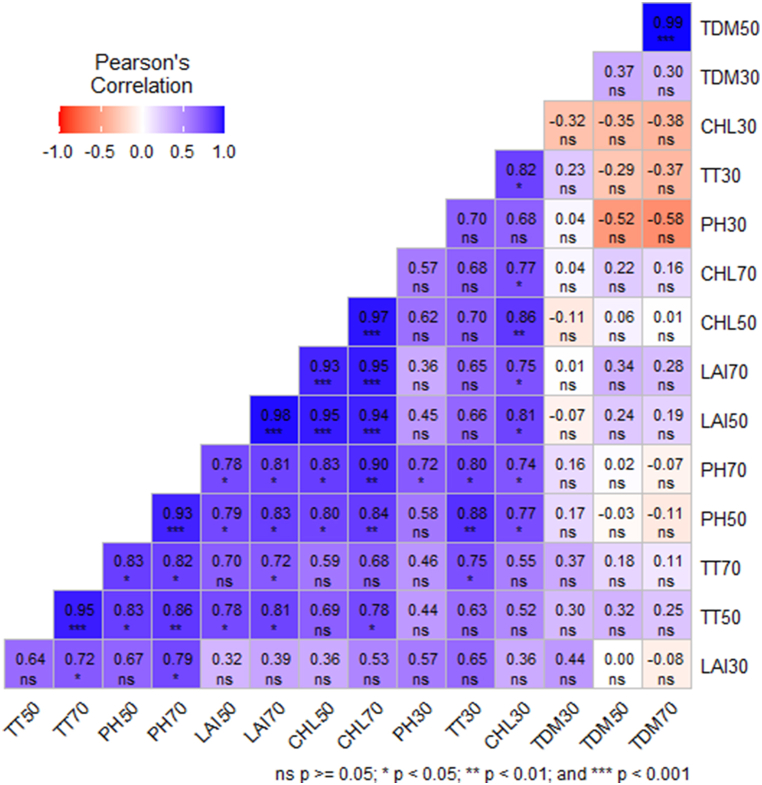
Pearson correlation analysis of vegetative growth traits in rice under the impact of oligo-chitosan on the growth of the widely cultivated variety, BRRI dhan29, in Bangladesh.Where PH30= Plant height at 30 DAT, PH50= Plant height at 50 DAT, PH70= Plant height at 70 DAT, TT30 = No. of total tiller hill^−1^ at 30 DAT, TT50 = No. of total tiller hill^−1^ at 50 DAT, TT70 = No. of total tiller hill^−1^ at 70 DAT, LAI30 = Leaf area index at 30 DAT, LAI50 = Leaf area index at 50 DAT, LAI70 = Leaf area index at 70 DAT, CHL30 = chlorophyll content at 30 DAT, CHL50 = chlorophyll content at 50 DAT, CHL70 = chlorophyll content at 70 DAT, TDM30 = Total dry matter at 30 DAT, TDM50 = Total dry matter at 50 DAT, TDM70 = Total dry matter at 70 DAT. ∗∗∗, ∗∗, ∗ and NS (Not Significant) represent probability of ≤0.001, ≤0.01, ≤0.05 and > 0.05, respectively.

A correlation was performed to identify the impact of oligo-chitosan on the growth of the widely cultivated variety, BRRI dhan29, in Bangladesh. (Fig. 11). The analysis revealed that number of chlorophyll content 70 at DAT had a strong positive correlation (<0.001) with chlorophyll content 50 at DAT, LAI at 50 and 70 DAT and moderately correlation with the PH at 50 and 70 DAT (P < 0.01) while with the TT at 50 DAT, it was corelated at P < 0.05. Chlorophyll content at 50 DAT had strong positive correlation with the LAI at 50 and 70 DAT besides with PH at 50 and 70 DAT it was corelated. LAI at 70 DAT demonstrated a strong positive correlation with the LAI at 50 DAT and corelated with the PH at 50 and 70 DAT, TT at 50 and 70 DAT. The PH at 50 and 70 DAT and TT at 50 DAT showed positive correlation with the LAI at 50 DAT. The PH at 70 DAT represented moderately positive correlation with TT at 50 DAT while corelated with the LAI at 30 DAT and TT at 70 DAT. The TT at 50 and 70 DAT correlated with the PH at 50 DAT. At 30 DAT chlorophyll content, pant height and LAI highlighted the non-significant negative correlation with the TDM 50 and 70 DAT while chlorophyll content at 30 DAT was with the TDM at 30 DAT.

**Fig. 12:**
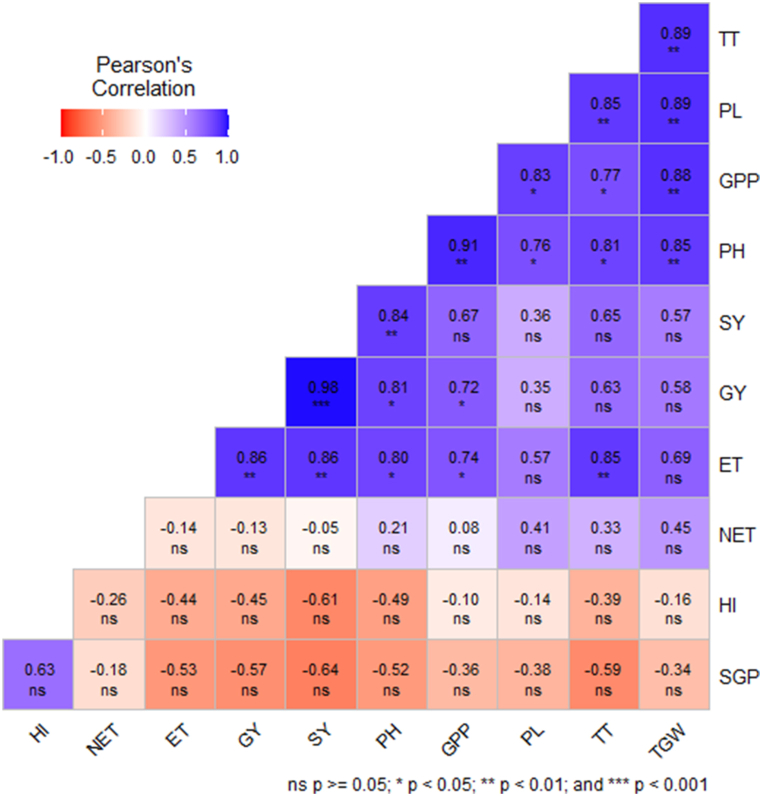
Pearson correlation analysis of yield and yield contributing traits in rice under the impact of oligo-chitosan on the growth of the widely cultivated variety, BRRI dhan29, in Bangladesh.Where PH= Plant height (cm), TT = Total tiller hill^−1^, ET = Number of effective tillers hill^−1^, NET= Number of non-effective tillers hill^−1^, PL= Panicle length (cm), GPP= Number of grain panicle^−1^, SGP= Sterile grains panicle^−1^, TGW = 1000-grain weight (g), GY = Grain yield (t ha^−1^), SY= Straw yield (t ha^−1^), and HI= Harvest index (%). ∗∗∗, ∗∗, ∗ and NS (Not Significant) represent probability of ≤0.001, ≤0.01, ≤0.05 and > 0.05, respectively.

When we come to the yield, and yield contributing character, the analysis revealed that the GY had a strong positive correlation (<0.001) with the SY and moderately correlated with the ET (P < 0.01) while the SY also moderately correlated with the ET (Fig. 12). The PH represented moderately positive correlation with SY while corelated with GY and ET (P < 0.05). The GPP demonstrated a moderately positive correlation with the PH and a correlation with the GY and ET. The PL was correlated with the PH and GPP. The number of TT showed a moderate mutualism with the ET and PL, and at the same time it maintained a correlation with the PH and GPP. Finally, TGW was represented a moderate positive correlation with PH, GPP, PL, and TT. So, this study revealed that oligo-chitosan had a positive impact on the growth and yield of the widely cultivated variety, BRRI dhan29, in Bangladesh.

#### 3.3.3 Heat map analysis

**Fig. 13:**
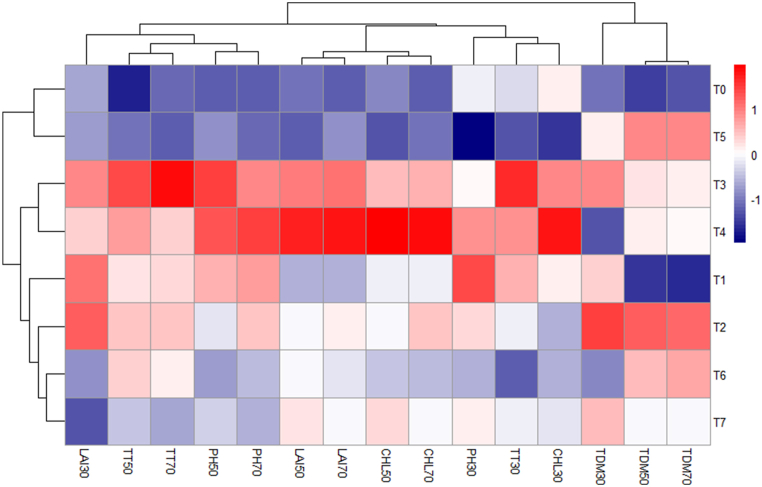
Heat map analysis of oligo-chitosan on the growth of the widely cultivated variety, BRRI dhan29, in Bangladesh. Where PH30= Plant height at 30 DAT, PH50= Plant height at 50 DAT, PH70= Plant height at 70 DAT, TT30 = No. of total tiller hill^−1^ at 30 DAT, TT50 = No. of total tiller hill^−1^ at 50 DAT, TT70 = No. of total tiller hill^−1^ at 70 DAT, LAI30 = Leaf area index at 30 DAT, LAI50 = Leaf area index at 50 DAT, LAI70 = Leaf area index at 70 DAT, CHL30 = chlorophyll content at 30 DAT, CHL50 = chlorophyll content at 50 DAT, CHL70 = chlorophyll content at 70 DAT, TDM30 = Total dry matter at 30 DAT, TDM50 = Total dry matter at 50 DAT, TDM70 = Total dry matter at 70 DAT. T_0_ = Control (no fertilizer and Chitosan), T_1_ = conventional method (with fertilizers), T_2_ = conventional method with foliar spray of 100 ppm chitosan solution, T_3_ = conventional method with foliar spray of 300 ppm chitosan solution, T_4_ = conventional method with foliar spray of 500 ppm chitosan solution, T_5_ = only foliar spray of 100 ppm chitosan solution, T_6_ = only foliar spray of 300 ppm chitosan solution, andT_7_ = only foliar spray of 500 ppm chitosan solution.

A heat map was used to analyze the traits that best demonstrate the impact of oligo-chitosan on the growth of the widely cultivated variety, BRRI dhan29, in Bangladesh. The x-axis represents the variables related to vegetative factors, while the y-axis represents the different treatments (Fig. 13). PH at 50,70 DAT, TT at 30, 50, 70 at DAT, chlorophyll content at 30, 50, 70 DAT, LAI at 30, 50, 70 DAT, and TDM at 30 DAT variables had a high contribution with conventional method and foliar spray of 300 ppm chitosan solution treatment. PH at 50,70 DAT, TT at 50, 70 at DAT, chlorophyll content at 50, 70 DAT, LAI at 30, 50, 70 DAT, and TDM at 30 DAT variables represented the lowest contribution with no fertilizer and Chitosan treatment. Other treatments-maintained light, moderate, and neutral relationships among all the parameters.

**Fig. 14:**
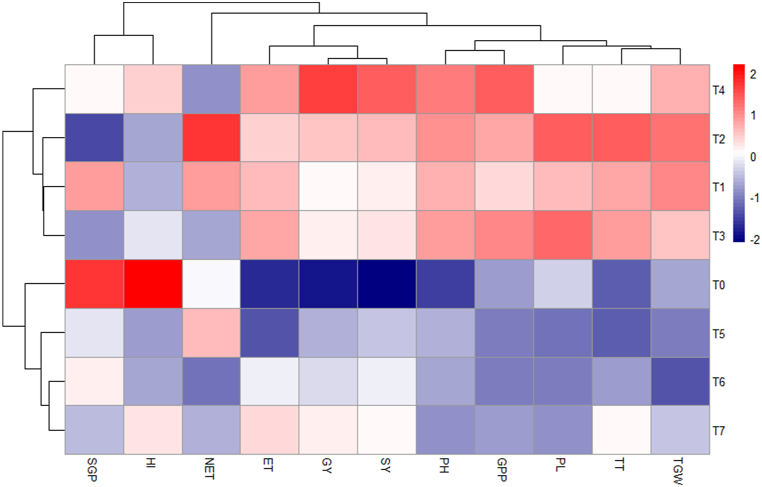
Heat map analysis of oligo-chitosan on the yield and yield contributing traits in rice under the impact of oligo-chitosan on the growth of the widely cultivated variety, BRRI dhan29, in Bangladesh.Where PH30= Plant height at 30 DAT, PH50= Plant height at 50 DAT, PH70= Plant height at 70 DAT, TT30 = No. of total tiller hill^−1^ at 30 DAT, TT50 = No. of total tiller hill^−1^ at 50 DAT, TT70 = No. of total tiller hill^−1^ at 70 DAT, LAI30 = Leaf area index at 30 DAT, LAI50 = Leaf area index at 50 DAT, LAI70 = Leaf area index at 70 DAT, CHL30 = chlorophyll content at 30 DAT, CHL50 = chlorophyll content at 50 DAT, CHL70 = chlorophyll content at 70 DAT, TDM30 = Total dry matter at 30 DAT, TDM50 = Total dry matter at 50 DAT, TDM70 = Total dry matter at 70 DAT. T_0_ = Control (no fertilizer and Chitosan), T_1_ = conventional method (with fertilizers), T_2_ = conventional method with foliar spray of 100 ppm chitosan solution, T_3_ = conventional method with foliar spray of 300 ppm chitosan solution, T_4_ = conventional method with foliar spray of 500 ppm chitosan solution, T_5_ = only foliar spray of 100 ppm chitosan solution, T_6_ = only foliar spray of 300 ppm chitosan solution, andT_7_ = only foliar spray of 500 ppm chitosan solution.

The x-axis represents the variables related to yield and yield contributing characters, while the y-axis represents the different treatments (Fig. 14). Yield and yield contributing character like GY, SY, PL, and GPP demonstrated the highest contribution when conventional method with foliar spray of 500 ppm chitosan solution was applied. Control (no fertilizer and Chitosan) treatment showed the lowest PH, GY, SY, and ET. Other treatments-maintained light, moderate, and neutral relationships among all the parameters.

## 4 Discussion

Rice is the most important cereal crop in Bangladesh which is planted around three-fourths of the cultivable land. Changing climate, decreasing agricultural land, and low yield of high yielding varieties in farmers' field limited the export of rice at international markets [48]. Moreover, excessive application of synthetic fertilizers to produce more causes negative impact on soil health and soil microorganisms’ biodiversity. Therefore, for sustainable crop production, there is a need to find alternatives or ways of reducing excessive fertilization without sacrificing rice yield potential. Chitosan is a natural biodegradable polymer that has been used in agriculture for its nontoxic, biocompatible, bio active, inherent antimicrobial, and eliciting properties [49]. Therefore, in our current study we investigated the potential of foliar spray of chitosan to improve rice yield and the results of our study clearly demonstrated that chitosan could be an effective option to achieve desirable high yield in rice production system.

Extracting the plant growth parameters has a great significance for predicting yield and improving the management of agricultural production [50,51]. Our results showed that foliar spray of chitosan with conventional system was able to increase PH, number of tillers hill^−1^ and LAI at different growth stages. A numerous study reported a positive effect of chitosan on PH, TT hill^−1^ and LAI on different crops [[52], [53], [54]]. Chitosan helps in the mineralization of organic nutrients into inorganic forms, which are easily absorbed by plant roots [55]. Thus, chitosan serves as a non-toxic and biodegradable plant growth promoter by enhancing performance of crop production. Moreover, chitosan have the ability to induce signal to synthesize plant hormones and pathways connected to auxin biosynthesis to enhance plants growth and development [56]. Plant treated with chitosan increases the amount of indole-3-acetic acid (IAA), which enhances cell division, and triggers the expression of a *PIN1* gene responsible for auxin translocation regulation [57]. Results of our study clearly indicated that chitosan spray increases the plants total dry weight. This is due to the additive effect of chitosan on plant growth and development in seedling stage that contribute to higher root and shoot growth of treated plants. The key to plants’ higher growth and yield is the high photosynthetic rate by which adequate supply of food from source to sink organ. About 16–29 % increase in chlorophyll content dependent on different growth stages was observed for the combined use of chitosan and conventional method compared to the conventional method. This clearly illustrates that chitosan can enhance the photosynthesis performance and the accumulation of organic matter in rice seedlings. Increase of chlorophyll content were also observed in rice [58], wheat [39], and maize [59] due to foliar application of chitosan. Among the different treatment combinations, foliar application of chitosan at different levels improved growth of rice plants to some extent compared to untreated control and conventional method. However, 500 ppm combined with conventional method seems to provide highest vegetative growth stimulating effect than untreated control and conventional method.

LAI, chlorophyll content, and TDM in the box plot measurements show how plant growth and development change over time in response to treatment. Conventional method with foliar spray of 300 ppm chitosan solution showed superior canopy development under this treatment, as evidenced by the fact that it consistently displayed the highest LAI across all intervals, especially at 50 DAT. Significant variations in the amount of chlorophyll between treatments were observed, which reflected different physiological reactions to treatments over time. Along with the rise in TDM accumulation over time, certain treatments showed higher biomass production, indicating improved resource use. Overall, the results demonstrate how crucial certain treatments are for maximizing the dynamics of plant growth [39,60].

The knowledge that is most of interest and important for grain production is crop yield and its yield contributing characters. The association between different economically advantageous traits to determine yield helps us to enhance productivity in a short time span. Findings of our study denoted an increased positive effect on yield contributing traits due to the foliar application of chitosan with conventional method. The foliar spray of 500 ppm chitosan showed the highest positive effect on PH, ET, GPP, and TGW traits also corroborating with the findings reported by other researchers [39,54,55]. In comparison to the single-use conventional method and control treatments, the combined application of 500 ppm chitosan with the conventional approach resulted in a substantial improvement in PH, ET, GPP, and straw production. These improvements demonstrate how chitosan works in concert to improve growth and yield statistics. Even lower chitosan concentrations may be useful, as indicated by statistically comparable outcomes for combinations of 100 and 300 ppm chitosan. Its function in decreasing wasteful growth and improving reproductive efficiency is further supported by the lowest levels of SGP and NET seen under 500 ppm chitosan spray. These findings demonstrate the potential of chitosan as a useful agronomic technique for raising rice yields [61]. The application of chitosan reduced the number of sterile GPP that meant the quality of rice grains was improved, which is consistent with the work of Boonlertnirun et al. [55]. Out of a few various concentrations tested in our current study, 500 ppm foliar spray of chitosan solution resulted in the maximum rice yield compared to the untreated control and conventional method. The potential of chitosan as a rice-growing treatment that increases yield. The efficiency of the 500-ppm chitosan spray is demonstrated by the notable yield increase that occurred both with and without the conventional technique. This shows that for sustainable rice cultivation, chitosan may be a viable substitute or addition to conventional methods [62]. The rice yield increase was about 25 % and 45 % higher than conventional method and control treatments, respectively when conventional method treatment was combined with 500 ppm foliar spray of chitosan solution. Several researchers reported that application of chitosan resulted in a yield increase of 21–31 % in rice [63], 13.6 % in wheat, and 20.5–39.8 % in maize [64]. Although our study does not unravel the mechanisms of growth and yield enhancement of rice by chitosan, however, chitosan is known to be involved in stimulating different physiological pathways which are involved in vegetative growth, followed by biosynthesis and active translocation of carbohydrates during stimulating cell division and forming DNA and RNA. In optimal production system, the increase in yield components may be because chitosan could enhance nitrogen and potassium in leaf, which lead to increase in the number of chloroplasts per cell, cell size, and number per unit area [65,66]. Consequently, it increased chlorophyll to improve photosynthesis rate and biomass, which was observed in our study. Proteomic analysis combined with co-expression network analysis on rice revealed that 90 % of the positively co-expressed chitosan-responsive genes for photosynthetic pigments enhancement were localized in chloroplast suggests that the chloroplast is a target organelle for chitosan action.

Conventional method with foliar spray of 300 ppm chitosan solution and conventional method with foliar spray of 500 ppm chitosan solution are strongly influenced by PC1, with variables like tiller number, LAI, and chlorophyll content, GY, and SY being highly correlated. In contrast, conventional method with foliar spray of 100 ppm chitosan solution and only foliar spray of 500 ppm chitosan solution aligns with PC2. Control (no fertilizer and Chitosan), conventional method (with fertilizers), and only foliar spray of 300 ppm chitosan solution has more balanced values near the origin [67]. At 50 and 70 DAT, there were strong positive correlations and tight alignments between the variables of chlorophyll content, LAI, and PH. However, at 30 DAT, there were non-significant negative associations found between TDM at 50 and 70 DAT and chlorophyll content, PH, and LAI [68]. Positive correlations between PH, ET, and GPP show that these factors affect GY together. Furthermore, PH, PL, and TT showed moderately positive correlations with TGW, indicating a relationship between these characteristics and increased production potential [69]. The heatmap analysis shows that while 500 ppm of chitosan foliar spray increased yield components including grain and SY, 300 ppm considerably improved plant growth characteristics. In comparison, the control treatment showed the lowest values across all tested parameters, indicating the benefits of chitosan and fertilizer applications [70].

Furthermore, application of chitosan significantly resulted in increase of SY. The increase of both grain and SY ultimately increases the HI. Compared to the single use of conventional method, additional supply of 500 ppm chitosan with conventional method increases around 20 % of SY. Higher vegetative growth in initial growth stages is correlated with higher photosynthetic rate which might contribute to higher economic yield [43,71]. Moreover, the enhance performance in terms of vegetative growth, GY, SY in rice could be as a fact that plants treated with chitosan are less liable to stress such as drought, salinity, low or high temperature [72].

According to this study, chitosan applied topically, particularly at 500 ppm, when combined with conventional methods, greatly improves rice growth, grain, and straw output. Better chlorophyll content, photosynthesis, nutrition uptake, and stress tolerance are some advantages of chitosan. As a sustainable substitute for use of synthetic fertilizers, the results point to chitosan. Future studies should investigate the underlying processes and evaluate how chitosan affects various environmental variables over the long run in order to maximize rice yield.

## 5 Conclusion

The results show that the rice cultivar BRRI dhan29 had an increase in vegetative parameters and several yield attributes when sprayed with chitosan along with conventional cultivation practice. We found that the conventional method with 500 ppm chitosan solution resulted in the highest grain and straw production in rice. In agricultural production systems, therefore, a chitosan formulation made from the naturally abundant chitin of crustaceans could be a great environmentally friendly agent for sustainable rice production, leading to an increase in rice output per unit area. However, to successfully commercialize chitosan application in rice and offer it as a production technique in Bangladesh, a thorough field study with other rice cultivars under varied environmental circumstances is required.

## CRediT authorship contribution statement

**Afrina Rahman:** Writing – original draft, Methodology, Formal analysis, Data curation. **Rayhan Ahammed:** Writing – original draft, Methodology, Formal analysis, Data curation. **Jayanta Roy:** Writing – original draft, Formal analysis. **Md Liton Mia:** Writing – original draft, Methodology, Formal analysis, Data curation. **Mohammad Abdul Kader:** Writing – review & editing. **Mubarak A. Khan:** Writing – review & editing, Supervision, Funding acquisition. **Md Harun Rashid:** Writing – review & editing, Validation. **Uttam Kumer Sarker:** Writing – review & editing, Software. **Md Romij Uddin:** Writing – review & editing, Funding acquisition. **Md Shafiqul Islam:** Writing – review & editing, Supervision, Methodology, Formal analysis, Data curation.

## Ethical statement

This study does not involve any human or animal subjects, and it is in accordance with research ethical standards.

## Data availability statement

All of the data are available in all Tables and Figures of the manuscripts.

## Funding statement

This research did not receive any specific grant from funding agencies in the public, commercial, or not-for-profit sectors.

## Declaration of competing interest

The authors declare that they have no known competing financial interests or personal relationships that could have appeared to influence the work reported in this paper.
