# Supplementary material for: Investigating the impact of oligo-chitosan on the growth dynamics and yield traits of Oryza sativa L. ‘BRRI dhan29’ under subtropical conditions
Source: Heliyon. 2024 Dec 28;11(1):e41552. doi: 10.1016/j.heliyon.2024.e41552 (PMC11751532; doi:10.1016/j.heliyon.2024.e41552)
Supplement: Multimedia component 1 [file mmc1.docx]

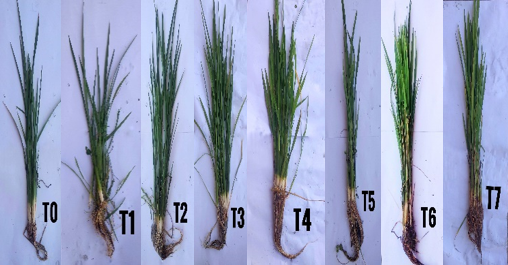


Supplementary Figure: Pictorial view of the BRRI dhan29 treated with different chitosan concentration. Here, Control (no fertilizer and Chitosan) (T_0_), conventional method (with fertilizers) (T_1_), conventional method with foliar spray of 100 ppm chitosan solution (T_2_), conventional method with foliar spray of 300 ppm chitosan solution (T_3_), conventional method with foliar spray of 500 ppm chitosan solution (T_4_), only foliar spray of 100 ppm chitosan solution (T_5_), only foliar spray of 300 ppm chitosan solution (T_6_), and only foliar spray of 500 ppm chitosan solution (T_7_).
